# Essentials for AI Research in Cardiology: Challenges and Mitigations

**DOI:** 10.1016/j.cjco.2024.07.015

**Published:** 2024-08-03

**Authors:** Biyanka Jaltotage, Girish Dwivedi

**Affiliations:** a Department of Cardiology, Fiona Stanley Hospital, Perth, Western Australia, Australia; b Harry Perkins Institute of Medical Research, Perth, Western Australia, Australia; c School of Medicine, The University of Western Australia, Perth, Western Australia, Australia

## Abstract

Technology using artificial intelligence (AI) is flourishing; the same advancements can be seen in health care. Cardiology in particular is well placed to take advantage of AI because of the data-intensive nature of the field and the current strain on existing resources in the management of cardiovascular disease. With AI nearing the stage of routine implementation into clinical care, considerations need to be made to ensure the software is effective and safe. The benefits of AI are well established, but the challenges and ethical considerations are less well understood. As a result, there is currently a lack of consensus on what the essential components are in an AI study. In this review we aim to assess and provide greater clarity on the challenges encountered in conducting AI studies and explore potential mitigations that could facilitate the successful integration of AI in the management of cardiovascular disease.

## Résumé

Les technologies utilisant l’intelligence artificielle (IA) sont florissantes, y compris dans le secteur de la santé. Le domaine de la cardiologie est en bonne position pour profiter de l’IA en raison de l’abondance de données et du manque de ressources pour prendre en charge les maladies cardiovasculaires. Comme l’IA se rapproche du stade où elle sera systématiquement intégrée aux soins cliniques, il est important de prendre en considération certains facteurs afin d’assurer l’utilisation efficace et sécuritaire des logiciels. Les avantages de l’IA sont bien établis, mais les problèmes et les considérations éthiques qu’elle soulève sont moins bien compris. Par conséquent, il n’existe pas de consensus actuellement sur les composantes essentielles d’une étude sur l’IA. Le but de cette analyse est d’évaluer et de mieux décrire les problèmes rencontrés lors de la réalisation d’études sur l’IA et d’explorer les facteurs d’atténuation possibles qui pourraient favoriser l’intégration efficace de l’IA à la prise en charge des maladies cardiovasculaires.

Artificial intelligence (AI) describes computational processes that simulate human intellect with the purpose of problem-solving.^1^ The concept was initially described by Alan Turing as early as the 1950s.^2^ Propelled by the processing power afforded by modern computing these concepts have now been transformed to reality, and the field of AI has experienced substantial advancements in recent years. These algorithms are now capable of analyzing vast amounts of data in only seconds with exceptional accuracy, and they are not vulnerable to the fatigue that is experienced by humans.^3^ Naturally, this makes AI attractive for data-heavy or repetitive tasks that are time-consuming. Recently, it has become evident that current health services are struggling to cope with the escalating demands of managing cardiovascular disease (CVD). The COVID-19 pandemic has underscored the strain on current health services in the management of CVD, a burden likely to intensify with the ongoing metabolic syndrome epidemic.^4^^,^^5^ Because CVD management is data-intensive, and also relies on multimodal data integration with waveform, imaging, and tabular data for clinical decision-making, AI applications are especially poised to handle these complexities.

Despite the excitement surrounding AI systems, a degree of caution is still needed. This is especially crucial in high-stakes health care environments like the management of CVD, where clinical decisions hold significant weight and outcomes can evoke emotive responses. Over the past few years there has been an explosion in the number of AI-related studies, with researchers developing algorithms to assist clinicians in improving speed and accuracy for a variety of tasks.^6^ Despite these benefits, it cannot be forgotten that AI brings with it unique challenges that need to be carefully considered and extensively critiqued before implementation into everyday clinical practice. Further understanding is therefore needed on how to analyze AI studies in cardiology. In this review we aim to improve the understanding of what constitutes the essentials of an AI study across health care, with a specific focus on the field of cardiology.

## AI

AI is an encompassing term referring to the development of systems or algorithms capable of advanced tasks such as learning, problem-solving, language understanding, and visual perception.^7^ Because of AI’s vast breadth of ability this technology is being seen in increasing applications affecting everyday life, and health care is no exception. In cardiology AI systems already exist that are being studied to improve arrhythmia detection, automate cardiac imaging analysis, and even to support clinicians for decision-making to guide coronary intervention.^7^ With such a breadth of systems in the pipeline an understanding of AI is necessary to appreciate how to evaluate these studies. Therefore, in this section although not comprehensive, we aim to provide a brief overview of the topic.

**Figure 1:**
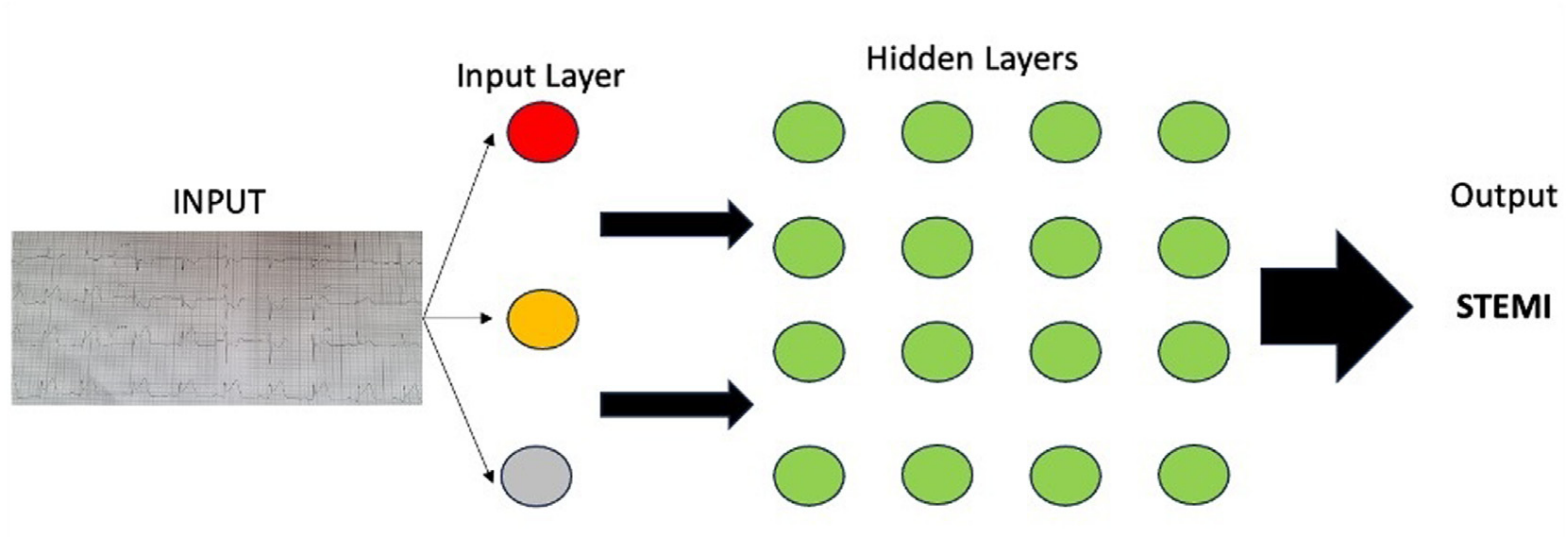
The framework of deep learning: an input such as an electrocardiogram is analyzed by a deep learning algorithm through a network of nodes. Each node requires a sufficient trigger to activate and then to trigger the subsequent nodes (hidden layers). This process concludes at the output layer which is then processed to provide an outcome to the end user. STEMI, ST-elevation myocardial infarction.

Machine learning (ML) lies at the core of AI; it is a subset of AI that describes computational and statistical algorithms frequently used to analyze large volumes of data. ML algorithms identify relationships between training data points not visible to humans, and with appropriate training accurate predictions on the basis of new previously unseen data is possible.^8^ Despite the effectiveness of ML, some tasks are better suited to deep learning (DL), a specialized subset of ML. The framework for DL consists of networks of nodes arranged in layers, the first layer also called the input layer when provided with a sufficient trigger is directed to a further layer called the hidden layer of which there might be multiple, and ultimately ending in the output layer (Fig. 1).^9^ This process is inspired by the physiological activation of neurons in the human brain. DL excels in the areas of image analysis, natural language processing, and speech recognition. Convolutional neural networks (CNNs) are the most frequently used DL models in health care. These CNNs predominantly process image-based data into layers of learnable weights and biases.^7^ The field of cardiology is dense with visual data and CNNs excel in these environments.

With the use of these AI techniques, systems are now capable of performing rapid analyses with exceptional accuracy. There is significant potential to enhance productivity to manage increasing workloads while preserving accuracy, ultimately improving outcomes for patients. Groups have been working on these algorithms for years and many are already in existence covering all aspects of cardiology. Automated electrocardiogram analysis tools using DL have the potential to be more accurate than senior cardiologists.^10^ Systems used in cardiac imaging have also shown competency in automating assessments of echocardiography, computed tomography, and magnetic resonance imaging.^7^ Furthermore, clinical decision support systems using ML have been shown to be effective in determining appropriate revascularization strategies using percutaneous coronary intervention.^11^ Considering this encouraging data, the increasing adoption of AI by various groups to address clinical challenges is not unexpected.

## Considerations and Challenges for an AI Study

With the potential for AI to revolutionize health care, a degree of caution with the uptake of the technology is rational. It is crucial to recognize that AI introduces its own set of challenges, which need to be carefully considered in all AI-related work to prevent negatively affecting patient care. Furthermore, awareness of these challenges is needed among readers of AI studies to understand if and how developers have addressed these challenges, which might influence the generalizability of the software. Outlining these challenges will ensure that algorithms are designed from the ground up to be effective, but also safe.

### Privacy and confidentiality

Systems using AI require access to a variety of data to produce accurate predictions. Frequently the data processing is then performed in a cloud-based setting, which requires sensitive data to be exported out of local facilities.^7^ The potential for data breaches exists in this process, yet confidentiality cannot be compromised in a quest to improve efficiency. Although some AI-based systems have already been implemented into routine clinical practice, AI in health care remains in its infancy and there are many more algorithms still in the pipeline. Therefore, it is essential that extensive measures are taken by developers to maintain end to end data confidentiality, before being contemplated for use in clinical practice. Approaches such as the use of generative AI models have potential by generating new synthetic data sets that resemble actual data sets, thereby nullifying the need for the system to hold onto sensitive patient data.^12^ Another proposed approach includes training AI systems from encrypted patient data such that the system is never directly exposed to patient data.^12^ Federated learning is another viable option to preserve data confidentiality. The fundamentals of federated learning involves a local copy of the relevant ML algorithm for users to use on their device.^13^ The local algorithm learns, and these new updated parameters are sent to a pooled centralized cloud server to improve the overall model. In this process the raw data are not exposed and remain on the local device. Developers will need to clearly outline what steps have been taken to convince users of the reliability of these methods. If systems are allowed that lack confidentially, patients and clinicians will lose confidence in the technology, and thus impede future developments.

### Algorithm bias

CVD, along with many other conditions, is common worldwide and affects individuals from all walks of life, regardless of culture or ethnicity. AI systems are entirely dependent on their training population for precision. Training populations that lack diversity are at high risk of underperforming with minority groups.^14^ If there is widespread adoption of these AI systems, suboptimal training populations could legitimately risk widening already existing health inequalities. Software bias against minority groups is already being reported, one such example is that of an AI-assisted model to automate segmentation of the ventricles and myocardium on cardiac magnetic resonance imaging. Accuracy was shown to be significantly different depending on patient race.^15^ Mitigating bias within AI systems entails implementing various strategies (Table 1). Incorporating built-in alarms that alert users when the system detects a high risk of bias could be a highly effective tool.^16^ Additionally, ensuring diversity within the training population helps counteract biases inherent in data sets, and subsequently specific testing can be then performed to confirm that algorithm performance remains high among minority groups. Furthermore, because of the dynamic nature of local populations because of global migration there will be a need for AI systems to remain up to date to sustain future accuracy and relevance. Developers should establish routine, predetermined intervals to assess the necessity for updates to the initial training populations. This will provide an assurance to users that the system continues to evolve in line with changing demographic characteristics. Lastly, training population diversity itself does not guarantee the absence of bias. In some situations, bias can be insidious and inherent to the design of the system. Obermeyer et al. discovered this by investigating a widely used algorithm to estimate patient risk to target patients for “high-risk management programs.”^17^ The authors reported that compared with White individuals, those who were Black had their risk significantly underestimated. Despite the richness and diversity of the data set it was reported that the bias stemmed from training data reliance on health costs as a surrogate for health needs. This error failed to recognize that Black individuals often faced barriers to access to health care, so despite lower health care costs the level of health among the Black population was poorer. Understanding the intricacies of bias in such circumstances requires transparency at every level of the algorithm design, which is unlikely to be made available by most commercial developers.

**Table 1:** Checklist for developers to address system bias

Checklist item	Description
Training population diversity	Algorithms need to be trained using diverse populations. A lack of diversity increases the risk of poor performance in minority groups
Subgroup performance testing	Specific testing of the algorithm should be performed in minority groups to ensure accuracy remains high in these populations
Built-in alarms	Developers should design alarm systems for users in scenarios at high risk of bias
Adaptations to changing demographic characteristics	A process needs to be established for system updates to incorporate factors such as changing demographic characteristics over time, ensuring the algorithm’s accuracy in the future
Transparency in algorithm design	Bias can be insidious and inherent to the algorithm. Therefore, a transparent software design provides a deeper understanding, which allows for the detection of design flaws at earlier stages

### Overfitting

A further challenge faced by AI studies relating to the training population is the concept of overfitting. As described earlier, the algorithms learn from the training data by identifying patterns, but this creates the risk of variations in the data being detected and extracted that are only specific to the training data set. Models that suffer from overfitting lack external applicability to the general population.^18^ The issue of overfitting needs to be suspected when a model performs increasingly well on the training data, but the same accuracy is not shown with the validation data set. Overfitting is most problematic with smaller training populations, but simply increasing the population might not always be feasible. Additionally, there is no widely agreed-upon or standardized minimum population size to mitigate overfitting. However, techniques do exist to overcome overfitting without having to increase the training cohort. With techniques such as data augmentation it is possible to artificially enrich the existing data set. Data augmentation allows the number of available data points to be expanded by introducing minor alterations to the original data set. For instance, with a set of images this could include cropping, flipping, rotation, or skewing to improve the diversity of the existing training data.^18^ Another strategy is transfer learning. This consists of using a model that has already been optimized from a large training data set, which is then redirected to a new task. Transfer learning works on the concept that some tasks might have similar processing requirements and that using an already existing system improves efficiency while reducing the risk of overfitting.^19^ With the use of similar techniques, all good AI studies need to take purposeful steps to address overfitting and improve the generalizability of their algorithms.

### The black box problem

All AI technology faces the challenges of the “black box problem,” and no simple solution exists. Explainability refers to the ability to grasp the general workings of a model’s decision-making process without examination of intricate details in a model.^20^ Frequently, explainability can be poor for AI systems because they are capable of connecting complex patterns in a way that no human ever could. The result is highly accurate predictions or outputs, but with minimal external explainability of how the answer was achieved.^21^ Ethically, the concept of the black box is challenging to overcome especially in health care. In medical decision-making, the “black box problem” becomes particularly sensitive, because many believe transparency and understanding are crucial to maintaining patient safety. This sensitivity is heightened in high-acuity areas fields like cardiology.

As previously mentioned, AI technology also holds the potential to exacerbate health disparities, with some of these disparities potentially obscured within the opaque nature of the black box. The algorithm could create outputs on the basis of confounding factors such as gender and race, which could be detrimental to overall population health. Gichoya et al. proved this concept by developing a DL model that was able to accurately predict race on the basis of medical images alone.^22^ Therefore, it is likely a degree of explainability is required in any AI study and might help to detect similar scenarios, but a requirement for complete explainability would hinder progress and performance.

**Figure 2:**
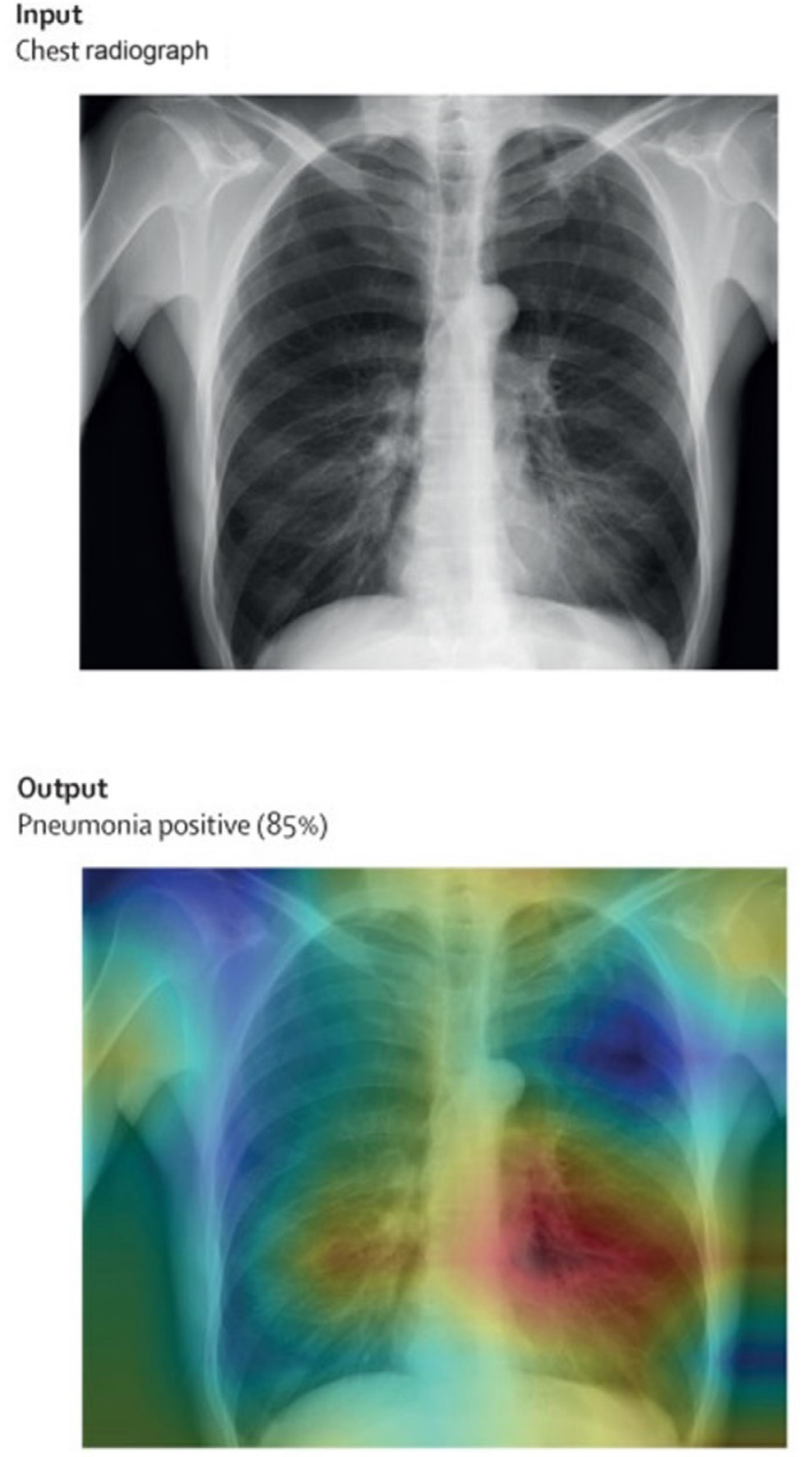
Explainable artificial intelligence with saliency maps: A chest radiograph is evaluated by an artificial intelligence system, and a heat map is used to improve explainability. In this case the system has produced an output with a likely diagnosis of pneumonia. The bottom saliency map image highlights in **red** the region of the image most critical to the final diagnosis Modified from Ghassemi et al.^23^ with permission.

**Figure 3:**
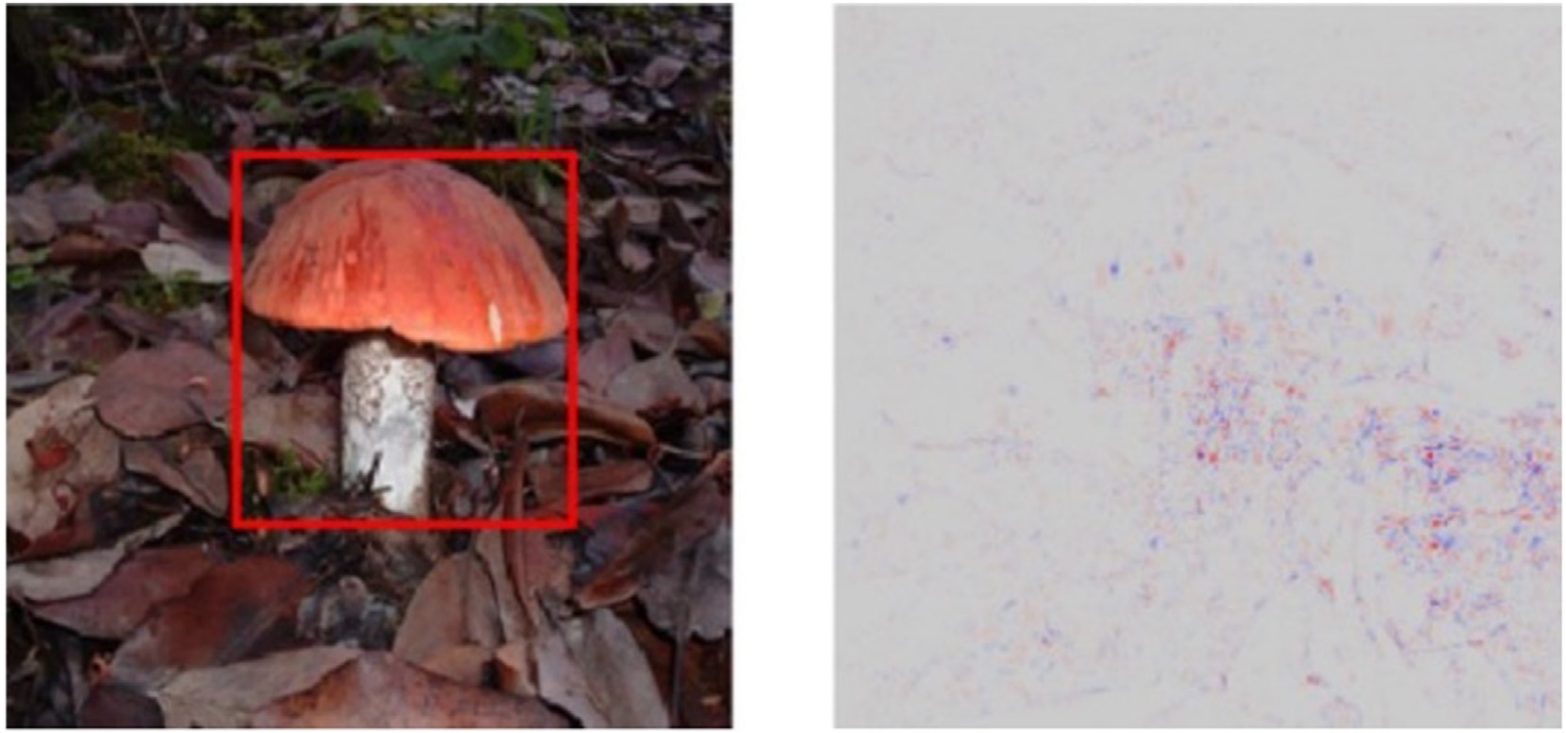
Feature visualization: feature visualisation technique identifying internal data patterns that were used by an algorithm to detect a mushroom in this image. Modified from Mohamed et al.^24^ with permission.

A number of techniques are now accepted to improve algorithm explainability. For example, saliency maps are an attractive visual solution, images such as chest radiographs showing pneumonia or heart failure could be colour graded depending on regions that are most critical to the system’s ultimate output (Fig. 2).^23^ The drawbacks of saliency maps are the risk of oversimplification of complex models, which could result in inaccuracies and the inherent limitations of this technique to only visual data. An alternative option is feature visualization, with this technique the model’s internal patterns are highlighted to better understand how a variety of inputs influence the outcome of the algorithm’s decision-making (Fig. 3).^24^ The challenges with this method include the complexity in interpreting the outputs, difficulties with scalability for large data sets, and a lack of flexibility with feature visualization, which performs best with visual data and CNNs. There are *post hoc* methods that are frequently used and 2 of the most established techniques are locally interpretable model-agnostic explanations and Shapley values. When applying locally interpretable model-agnostic explanations, precise adjustments are made to specified inputs to discern which inputs are most significantly changing outputs.^23^ Likewise, Shapley values provide a standardized method to ascertain the fractional contributions of a wide range of inputs.^25^ The shortcomings of these *post hoc* methods are the complexity, computational intensiveness for large data sets, and a lack of generalizability across the entire data set because they only provide local explanations.

Despite the recent advancements made in the field of explainability it is likely that shifting the focus to interpretable AI rather than explainability will yield the greatest benefits. The key difference between interpretable and explainable AI is that interpretable systems are transparent in design without the need to apply further tools or analysis to improve comprehension. Explainability requires additional analysis and techniques to make it explainable. Systems that have been built to be inherently interpretable are also likely to further improve accuracy.^26^ A fixation on explainability can also prove to be detrimental because explainable models are not entirely faithful to the original models, which might lead to errors with potentially significant consequences.^26^ Interpretable systems enable human understanding of the inner workings of a model, including insight into the final output. This approach has been supported by the Food and Drug Administration (FDA) in their 2021 action plan as part of the good ML practices.^27^ Interpretability is seen best in cases that are designed from the ground up by developers with this idea in mind. However, interpretable models will not be needed in all scenarios. Lower-risk decisions such as a system used to create patient appointments would not require a high level of interpretability. Similarly, the need for interpretability is less stringent if clinicians can verify and modify system output; an example could be a ML model that quantifies the severity of valvular lesions on echocardiography, which is then subsequently reviewed by an imaging specialist.^28^ Conversely, AI-assisted robotic coronary intervention is a high-risk environment and would require interpretability to allow any errors to be understood and corrected.

### Ethical considerations

Despite the widespread opportunities for benefits with the implementation of AI in cardiology, clinicians still have their reservations. Many of these reservations surround ethical challenges presented by the technology. The concepts of privacy and confidentiality, algorithm bias, and the black box problem discussed previously all raise ethical concerns. In addition, there are further ethical concerns to be considered. For example, with the volumes of data being input into these systems data ownership rights become unclear. Furthermore, with the use of AI-assisted decision-making questions arise with regards to liability. If and when medical errors occur, will the liability be with the user or software manufacturer, or would there be a concept of “shared liability”?^29^ Last, there are societal concerns that need to be tempered surrounding automated systems. Most surveys of the general public have shown that automated decision-making without any human input is not supported in health care environments.^30^ This highlights the importance of ongoing clinician involvement despite the availability of AI algorithms.

## Implementation of AI

The underpinnings of AI technology can be complex, and only individuals with specialist knowledge in the field will have a comprehensive understanding. Therefore, if these algorithms were to be incorporated into everyday clinical practice, most users would lack this specialized knowledge. Moreover, it would be unreasonable to expect all users to have intimate knowledge of AI. Consequently, good AI systems will therefore need to focus on ease of use. A large aspect of this will be the development of an approachable user interface without a significant learning curve. Interfaces that are easily learnable will encourage the uptake of the technology, while also minimizing the opportunity for user error, which could adversely affect patients. Furthermore, it will be key for AI studies to have a strong understanding of how their systems can fit into existing workflows. The more seamless the integration, the more success developers could have with end users incorporating the use of the software into their routine clinical practice. Another consideration for smooth integration is the minimum hardware requirements. AI-based systems frequently demand substantial processing power, potentially limiting overall usability. Hence, it is essential that AI studies should also emphasize accessibility, by optimizing system efficiency thereby reducing the need for major overhauls of current hardware.

Ensuring the appropriate use of AI models as intended is paramount. There might be a temptation for users to extend the application of such systems beyond their original scope, posing a risk to patients. To address this concern the concept of “model facts” was introduced.^31^ It aims to inform end users about the appropriate circumstances for using an AI system in clinical decision-making, as well as when not to do so. It has been proposed that “model facts” should accompany the release of any AI software intended for clinical use as standard practice. Educating users through such methods will maximize the potential benefits of each system while minimizing the risks.

AI developers also encounter a myriad of challenges when crafting AI systems for health care applications. Similarly, governing bodies shoulder immense responsibility in scrutinizing such software to guarantee its safety and suitability for deployment. Consequently, several expert panels have contributed their insights to aid successful AI integration.^32^, ^33^, ^34^ In line with these efforts, our group has pioneered a traffic light system, striving to provide further clarity in this complex landscape (Table 2). Furthermore, updates have been made to the Consolidated Standards of Reporting Trials (CONSORT) and Standard Protocol Items: Recommendations for Interventional Trials (SPIRIT) Guidelines, which provide evidence-based recommendations to improve the reporting of randomized controlled trials and clinical trial protocols, respectively. The updates have focused on addressing AI and have culminated in the establishment of the CONSORT-AI and SPIRIT-AI guidelines.^35^^,^^36^ With more accessible resources, developers and reviewers of novel AI solutions have more guidance to optimize safety and performance. Because of the rapid evolution within the field and the potential for unforeseen challenges, regular reassessment of AI guideline documents is likely to be imperative in the years ahead.

**Table 2:** Proposed traffic light system checklist for an AI algorithm

		
Purpose		
	Describe the objective of the algorithm and why an approach with AI is needed	
	Clearly define the task of the algorithm and its scope	
Design		
	Describe the data sets used in detail, with explanations of why they were chosen and specifically the diversity of the data set. This includes the validation data set	
	Detail how the data were prepared to preserve confidentiality but also to remain clean and consistent. Include if data augmentation or similar techniques were used	
	What data records and inputs were required to develop the algorithm?	
	Outline the completeness of the original data set and the values that were missing and how these were managed. Include any techniques to manage outliers	
	Describe the AI techniques used to develop the algorithm and why	
	Who were the team members involved in the development of the algorithm and did they have a wide range of expertise and background?	
	Detail the steps taken to ensure the technology can be integrated into existing health care systems. What is the expected workflow?	
	How has the usability of the AI system and its interface been optimized and assessed?	
Robustness		
	Describe the accuracy of the system and any outcome data. How does it compare with systems that are currently in place?	
	Has the intraobserver and interobserver variability been described?	
	What steps have been taken to optimize the performance of the algorithm?	
	Are there alerts inbuilt to warn users when levels of certainty fall below threshold? How was certainty defined and how was the threshold chosen?	
	Describe the mechanisms in place to monitor performance, and for the system to adapt over time to ensure accuracy does not wane	
	What considerations and techniques have been used to prevent overfitting?	
Ethical considerations		
	Outline the techniques used to provide explainability. How has the degree of explainability of the algorithm been assessed?	
	What steps have been taken to identify and address bias?	
	How has patient data been secured to maintain confidentiality?	
	How transparent is the algorithm code to allow for reproducibility?	

Each point should be assessed by an independent body, if appropriate considerations have been made a green light is given. If considerations do not pass, then a red light is given, and the system is not passed into clinical use. A yellow light can be given if the system could be approved with modifications.

AI, artificial intelligence.

Modified from Jaltotage et al.^7^ with permission.

Overall, the regulatory landscape for AI in health care is unique to other regimes and remains underdeveloped.^37^ Developers need to be aware of the documents produced by expert panels to ensure their AI algorithms are designed and implemented in a manner to minimize ethical concerns and also maximize the opportunity for clinical success. A stepwise approach such as the traffic light checklist could provide the blueprint for the necessary structure for developers and regulators (Table 2). Similarly, the FDA, Health Canada, and the United Kingdom’s Medicine and Healthcare products Regulatory Agency jointly established 10 guiding principles for developers to navigate good ML practices.^38^ The principles highlight areas such as workflow integration, data quality assurance, robust cybersecurity, and results generalizable to the population of interest in real world settings. Together these 10 principles currently form the basis to evaluate health care-related AI technology. Consensus among major regulatory bodies provides blueprints to developers and is a large step in the right direction to achieving responsible AI implementation. As the field continues to evolve further updates are inevitable, and regulatory bodies have shown a commitment to openness by encouraging discussion and actively seeking feedback from stakeholders.^39^ The FDA offers publicly available lists of AI enabled medical devices that have successfully met all premarket requirements, and at the time of writing 171 devices have been included on the published list.^40^ A review in 2021 of this list revealed that despite FDA approval most medical AI devices had only undergone retrospective study at a small number of sites. Furthermore, almost half of all published reports did not include sample sizes, and of those that did the median sample size was modest at approximately 300.^41^ Since that time the 10 guiding principles have been introduced, which do highlight and address these shortcomings. However, this underscores the ongoing need for regulatory innovation in parallel with advancements in AI-related technology to optimize overall performance and patient safety.

## Conclusion

The field of AI has made substantial advancements in recent years. It seems inevitable now that this technology will affect everyday life, including health care. The speciality of cardiology, in particular, stands to gain significantly because it is data-heavy, can be repetitive, and consists of an array of image-based tests where AI can excel. Despite the potential benefits, AI brings with it unique challenges that need to be addressed for it to be successful in the health care space. There is also no current consensus on the key components of a good AI study to guide designers of these algorithms. Because of the current state of the knowledge base, further work is still needed to establish a universal understanding of the essential elements that constitute a robust AI study.

## Declaration of Generative AI and AI-Assisted Technologies in the Writing Process

During the preparation of this work the author used ChatGPT (openAI, San Francisco, CA) to improve the readability and language. After using ChatGPT, the authors reviewed and edited the content as needed and take full responsibility for the content of the publication.

## References

[1] Géron A. (2022).

[2] Epstein R., Roberts G., Beber G. (2009).

[3] Jaltotage B., Sukudom S., Ihdayhid A.R., Dwivedi G. (2023). Enhancing risk stratification on coronary computed tomography angiography: the role of artificial intelligence. Clin Ther.

[4] Eckel R.H., Grundy S.M., Zimmet P.Z. (2005). The metabolic syndrome. Lancet.

[5] Hirode G., Wong R.J. (2020). Trends in the prevalence of metabolic syndrome in the United States, 2011-2016. JAMA.

[6] Panch T., Mattie H., Celi L.A. (2019). The “inconvenient truth” about AI in healthcare. NPJ Digit Med.

[7] Jaltotage B., Ihdayhid A.R., Lan N.S. (2023). Artificial intelligence in cardiology: an Australian perspective. Heart Lung Circ.

[8] Johnson K.W., Torres Soto J., Glicksberg B.S. (2018). Artificial intelligence in cardiology. J Am Coll Cardiol.

[9] Sehly A., Jaltotage B., He A. (2022). Artificial intelligence in echocardiography: the time is now. Rev Cardiovasc Med.

[10] Zhu H., Cheng C., Yin H. (2020). Automatic multilabel electrocardiogram diagnosis of heart rhythm or conduction abnormalities with deep learning: a cohort study. Lancet Digit Health.

[11] Davies J.E. (September 21-25, 2018).

[12] Murdoch B. (2021). Privacy and artificial intelligence: challenges for protecting health information in a new era. BMC Med Ethics.

[13] Rahman A., Hossain M.S., Muhammad G. (2023). Federated learning-based AI approaches in smart healthcare: concepts, taxonomies, challenges and open issues. Cluster Compu.

[14] Lohr S. (2022). Ethics of Data and Analytics.

[15] Puyol-Antón E., Ruijsink B., Mariscal Harana J. (2022). Fairness in cardiac magnetic resonance imaging: assessing sex and racial bias in deep learning-based segmentation. Front Cardiovasc Med.

[16] Parikh R.B., Teeple S., Navathe A.S. (2019). Addressing bias in artificial intelligence in health care. JAMA.

[17] Obermeyer Z., Powers B., Vogeli C., Mullainathan S. (2019). Dissecting racial bias in an algorithm used to manage the health of populations. Science.

[18] Mutasa S., Sun S., Ha R. (2020). Understanding artificial intelligence based radiology studies: what is overfitting?. Clin Imaging.

[19] Kim D., MacKinnon T. (2018). Artificial intelligence in fracture detection: transfer learning from deep convolutional neural networks. Clin Radiol.

[20] Petch J., Di S., Nelson W. (2022). Opening the black box: the promise and limitations of explainable machine learning in cardiology. Can J Cardiol.

[21] Wang F., Kaushal R., Khullar D. (2020). Should health care demand interpretable artificial intelligence or accept “black box” medicine?. Ann Intern Med.

[22] Gichoya J.W., Banerjee I., Bhimireddy A.R. (2022). AI recognition of patient race in medical imaging: a modelling study. Lancet Digit Health.

[23] Ghassemi M., Oakden-Rayner L., Beam A.L. (2021). The false hope of current approaches to explainable artificial intelligence in health care. Lancet Digit Health.

[24] Mohamed E., Sirlantzis K., Howells G. (2022). A review of visualisation-as-explanation techniques for convolutional neural networks and their evaluation. Displays.

[25] Srinivasu P.N., Sandhya N., Jhaveri R.H., Raut R. (2022). From blackbox to explainable AI in healthcare: existing tools and case studies. Mobile Information Systems.

[26] Rudin C. (2019). Stop explaining black box machine learning models for high stakes decisions and use interpretable models instead. Nat Mach Intell.

[27] US Food & Drug Administration Artificial Intelligence/Machine Learning (AI/ML)-Based Software as a Medical Device (SaMD) Action Plan. https://www.fda.gov/media/145022/download.

[28] Rudin C., Chen C., Chen Z. (2022). Interpretable machine learning: fundamental principles and 10 grand challenges. Statistic Surveys.

[29] D’Antonoli T.A. (2020). Ethical considerations for artificial intelligence: an overview of the current radiology landscape. Diagn Interv Radiol.

[30] Araujo T., Helberger N., Kruikemeier S., De Vreese C.H. (2020). In AI we trust? Perceptions about automated decision-making by artificial intelligence. AI & Society.

[31] Sendak M.P., Gao M., Brajer N., Balu S. (2020). Presenting machine learning model information to clinical end users with model facts labels. NPJ Digit Med.

[32] Hanneman K., Playford D., Dey D. (2024). Value creation through artificial intelligence and cardiovascular imaging: a scientific statement from the American Heart Association. Circulation.

[33] Slart R.H., Williams M.C., Juarez-Orozco L.E. (2021). Position paper of the EACVI and EANM on artificial intelligence applications in multimodality cardiovascular imaging using SPECT/CT, PET/CT, and cardiac CT. Eur J Nucl Med Mol Imaging.

[34] Tang A., Tam R., Cadrin-Chênevert A. (2018). Canadian Association of Radiologists white paper on artificial intelligence in radiology. Can Assoc Radiol J.

[35] Liu X., Rivera S.C., Moher D. (2020). Reporting guidelines for clinical trial reports for interventions involving artificial intelligence: the CONSORT-AI extension. Lancet Digit Health.

[36] Rivera S.C., Liu X., Chan A.W. (2020). Guidelines for clinical trial protocols for interventions involving artificial intelligence: the SPIRIT-AI extension. Lancet Digit Health.

[37] Vokinger K.N., Feuerriegel S., Kesselheim A.S. (2021). Continual learning in medical devices: FDA’s action plan and beyond. Lancet Digit Health.

[38] US Food & Drug Administration Good Machine Learning Practice for Medical Device Development: Guiding Principles. https://www.fda.gov/media/153486/download.

[39] US Food & Drug Administration Using Artificial Intelligence and Machine Learning in the Development of Drug and Biological Products: Discussion Paper and Request for Feedback. https://www.fda.gov/media/167973/download.

[40] US Food & Drug Administration Artificial Intelligence and Machine Learning (AI/ML)-Enabled Medical Devices. https://www.fda.gov/medical-devices/software-medical-device-samd/artificial-intelligence-and-machine-learning-aiml-enabled-medical-devices.

[41] Wu E., Wu K., Daneshjou R. (2021). How medical AI devices are evaluated: limitations and recommendations from an analysis of FDA approvals. Nat Med.

